# Methiothepin Suppresses Human Ovarian Cancer Cell Growth by Repressing Mitochondrion-Mediated Metabolism and Inhibiting Angiogenesis In Vivo

**DOI:** 10.3390/pharmaceutics12070686

**Published:** 2020-07-20

**Authors:** Jin-Young Lee, Changwon Yang, Whasun Lim, Gwonhwa Song

**Affiliations:** 1 Department of Pharmacology and Toxicology, Medical College of Wisconsin, Milwaukee, WI 53226, USA; jylee@mcw.edu; 2 Institute of Animal Molecular Biotechnology and Department of Biotechnology, Korea University, Seoul 02841, Korea; ycw117@korea.ac.kr; 3 Department of Food and Nutrition, Kookmin University, Seoul 02707, Korea

**Keywords:** methiothepin, ovarian cancer, mitochondria, paclitaxel

## Abstract

Ovarian cancer is the fifth leading cause of cancer-related deaths in women. Despite treatment, most patients experience relapse and the 5-year survival rate of ovarian cancer is less than 50%. Serotonin has cell growth-promoting functions in a variety of carcinomas, but the effect of serotonin receptor antagonists on ovarian cancer cells is unknown. In this study, it was confirmed that methiothepin, a serotonin receptor antagonist, suppresses the viability of, and induces apoptosis in, ovarian cancer cells. Methiothepin also induces mitochondrial dysfunction, represented by depolarization of the mitochondrial membrane and increased mitochondrion-specific Ca^2+^ levels, and causes metabolic disruption in cancer cells such as decreased ATP production and oxidative phosphorylation. Methiothepin also interferes with vascular development in transgenic zebrafish embryos. Combination treatment with methiothepin improves the anti-cancer effect of paclitaxel, a standard chemotherapeutic agent. In conclusion, this study revealed that methiothepin is a potential novel therapeutic agent for ovarian cancer treatment.

## 1. Introduction

Ovarian cancer is the second most frequent and most lethal gynecological malignancy [1]. It has a 5-year survival rate of approximately 40%, ranking fifth with regard to cancer-related deaths among women [2]. The currently accepted treatment for ovarian cancer is the surgical approach followed by paclitaxel- and platinum-based chemotherapy. However, unfortunately, most cases of ovarian cancer recur because of chemoresistance; thus, the development of an agent that can support existing chemotherapy is required [3]. About 70% of ovarian cancers are resistant to chemotherapy [4]. The mechanisms underlying the progression and drug resistance in ovarian cancer are still not well understood.

In ovarian cancer, G protein coupled receptor (GPCR)-dependent Ca^2+^ signaling is reported to be activated by many types of neurotransmitters [5]. Alteration in the signaling cascades by activation of GPCRs contributes to cell proliferation, angiogenesis, and metastasis in various cancer types including ovarian cancer [6]. Serotonin (5-hydroxytryptamine) is a neurotransmitter that regulates mood and plays an important role in homeostasis and intestinal motility. Selective serotonin-reuptake inhibitors (SSRIs) are used clinically as antidepressants. In addition to its endocrine effects, serotonin acts as a cell growth regulator [7]. Serotonin has a proliferation-promoting effect in various carcinomas such as breast cancer, colon cancer, and lung cancer [8]. Serotonin also has a mitogenic effect in ovarian cancer cells [9]. The expression of serotonin receptors in ovarian cancer has also been verified by immunohistochemistry [10]. SSRIs may reduce the risk of ovarian cancer, suggesting that they may serve as a new chemotherapeutic approach for this disease [11]. 

There is some evidence that regulation of serotonin activity can inhibit the progression or increase the drug sensitivity of ovarian cancer, but little is known about how serotonin antagonists affect ovarian cancer cells. Methiothepin is a well-known non-selective 5-HT antagonist. Although little is known about the physiological activity of methiothepin in human cells, our previous study revealed that methiothepin can inhibit the proliferative potential and induce the death of prostate cancer cells [12]. Therefore, this study evaluated alterations in the proliferation and viability of the ovarian cancer cell lines ES2 and OV90 in response to methiothepin treatment. We also investigated whether methiothepin could induce mitochondrial disruption and metabolic imbalances in ovarian cancer cells. In addition, we analyzed the inhibitory effect of methiothepin on vascular formation using zebrafish embryos. Finally, the synergistic anti-cancer effect of methiothepin on ovarian cancer cells was verified through combination treatment with paclitaxel, a standard drug in chemotherapeutic approaches for the treatment of ovarian cancer. 

## 2. Materials and Methods

### 2.1. Reagents

Methiothepin was purchased from Sigma-Aldrich (St.Louis, MO, USA). Z-VAD FMK was purchased from Sigma-Aldrich. Antibodies against MCL-1, BCL-2, BCL-XL, PARP, GRP78/Bip, phosphorylated PERK (p-PERK), ATF4, and CHOP were obtained from Cell Signaling Technology (Danvers, MA, USA). Paclitaxel was purchased from Sigma-Aldrich. 

### 2.2. Cell Culture

ES2 and OV90 cells were obtained from the American Type Culture Collection (ATCC). Cells were grown in McCoy’s 5A Medium with 10% fetal bovine serum (FBS). Cells were seeded into culture dishes 24 h prior to the chemical treatments. Dimethyl sulfoxide (at the same volume as methiothepin) was used as the vehicle control.

### 2.3. Cell Viability and Proliferation Test

A trypan blue exclusion assay was performed to evaluate the viability and proliferation of ES2 and OV90 cells. To quantify the number of cells, counting was done with a hemocytometer on a brightfield microscope. In detail, cell viability was calculated as the ratio of living cells to total cells, the sum of living and dead cells, and cell proliferation was calculated as the proportion of living cells. In addition, a Cell Titer-Glo Luminescent assay was performed to analyze the number of viable cells based on adenosine triphosphate (ATP) production. 

### 2.4. Morphological Analysis

For morphological analysis, ES2 and OV90 cells were treated with methiothepin for 48 h. Cell morphology was assessed using Differential Quik (Diff-Quik) staining (Polysciences, Warrington, PA, USA).

### 2.5. Spheroid Culture

For spheroid formation, ovarian cancer cells were aliquoted on a round-bottom plate. Images of cells cultured for seven to 10 days with methiothepin were converted to 3D graphics using ReViSP software (The MathWorks, Inc., Natick, MA, USA).

### 2.6. Apoptosis Analysis

Propidium iodide (PI) and Hoechst 33342 dyes were used to identify apoptotic cells by nucleus fragmentation and condensation. We also performed Annexin V and PI staining, which were estimated by flow cytometry (BD Biosciences, Franklin Lakes, NJ, USA). ES2 and OV90 cells treated with methiothepin were incubated in a buffer containing 5% of Annexin V and PI, respectively. Cells were analyzed using a suitable excitation laser, calibrated to prevent channel interference. To analyze the activity of caspase, a Caspase-Glo 3/7 assay was used.

### 2.7. Western Blotting

The concentration of protein extracted from ovarian cancer cells was measured by the Bradford protein assay. Proteins resolved based on size using SDS-PAGE were transferred onto a nitrocellulose membrane. The blots were captured by chemiluminescent detection. The expression intensities of the target proteins were normalized using the expression intensity of β-actin.

### 2.8. Analysis of Mitochondrial Membrane Potential

Mitochondrial membrane potential (ΔΨ) was estimated using a Mitochondrial Staining Kit (Sigma-Aldrich) according to the manufacturer’s instructions. ES2 and OV90 cells treated with methiothepin for 24 h were stained with JC-1 dye for 20 min. Fluorescence intensity of the stained cells was measured using a flow cytometer.

### 2.9. Mitochondrial Calcium Analysis

Mitochondrion-specific Ca^2+^ levels were analyzed using Rhod-2 AM (Invitrogen). ES2 and OV90 cells treated with methiothepin for 24 h were stained with Rhod-2 AM dye for 30 min. Fluorescence intensity in the stained cells was measured using a flow cytometer.

### 2.10. Seahorse Analysis

For the mitochondrial stress analysis, oligomycin, carbonyl cyanide-4-(trifluoromethoxy) phenylhydrazone (FCCP), rotenone, and antimycin were injected in sequence. A Mitochondrial Stress Analysis Kit (Agilent Technologies) was used to evaluate the levels of metabolic indicators.

### 2.11. Angiogenesis Validation In Vivo

For angiogenesis validation, we maintained zebrafish as described previously [13], and treated them with methiothepin at the indicated concentrations. The effects of methiothepin on blood vessel formation were evaluated by Fli-tagged GFP fluorescence signals.

### 2.12. Statistical Analysis

Significant differences between the control and methiothepin-treated groups were evaluated by one-way or two-way ANOVA. These were defined as statistically significant when the p-values were less than 0.05. All experiments were conducted independently at least thrice.

## 3. Results

### 3.1. Methiothepin Inhibits the Viability of Ovarian Cancer Cells

**Figure 1:**
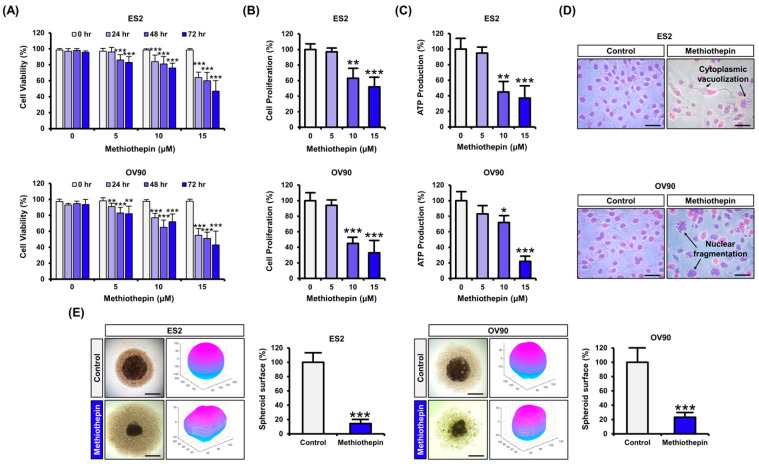
Methiothepin inhibits the viability and proliferation of ES2 and OV90 cells. (**A**) The viability of ES2 and OV90 cells, in accordance with the dose and duration of treatment with methiothepin, was analyzed by trypan blue exclusion assay. (**B**) Changes in proliferation of ES2 and OV90 cells following methiothepin treatment were analyzed by trypan blue exclusion assay. (**C**) The effect of methiothepin on intracellular ATP levels in ES2 and OV90 cells was assessed via colorimetric analysis. (**D**) Morphological changes of ES2 and OV90 cells following methiothepin treatment were analyzed by Diff-Quik staining. (**E**) The 3D structure for spheroid formation of ES2 and OV90 cells following methiothepin treatment was quantified using the ReViSP software. Results are expressed as the mean ± SDs of three independent experiments. The asterisks indicate statistically significant differences compared to the control (* *p* < 0.05; ** *p* < 0.01; *** *p* < 0.001). Scale bar indicates 100 μm.

To determine whether methiothepin has an anti-cancer effect on ovarian cancer cells, we tested the dose- (0, 5, 10, and 15 μM) and time-dependent (0, 24, 48, and 72 h) effects of methiothepin on the ovarian cancer cell lines ES2 and OV90. After analyzing the change in the viability of ovarian cancer cells in response to methiothepin, it was confirmed that the cell viability was significantly reduced in a dose- and time-dependent manner (Figure 1A). LC_50_ concentrations at 72 h of methiothepin treatment were determined at 15.31 μM in ES2 cells and 14.01 μM in OV90 cells. We set the treatment with 15 μM methiothepin for 24 h as the optimal treatment condition for subsequent experiments. In addition, methiothepin inhibited proliferative capacity in ES2 and OV90 cells in a dose-dependent manner (0, 5, 10, and 15 μM) (Figure 1B). In addition, methiothepin dose-dependently (0, 5, 10, and 15 μM) inhibited ATP production in ovarian cancer cells, suggesting that it disrupted the energy homeostasis (Figure 1C). Morphological analysis revealed that exposure to methiothepin damages the morphology of ovarian cancer cells, compared to that of the control (Figure 1D). In ES2 cells, morphological changes appeared mainly in the form of cytoplasmic vacuolization. Meanwhile, in OV90 cells, methiothepin treatment was found to induce nuclear fragmentation. Next, we formed spheroids to subject the cells to steric methiothepin exposure. When treated with 15 μM methiothepin for 24 h, the surface area of cells was significantly reduced (Figure 1E). These results suggest that treatment with methiothepin in ovarian cancer cells may decrease their viability, along with morphological alterations.

### 3.2. Methiothepin Induces Apoptotic Cell Death in Ovarian Cancer Cells

**Figure 2:**
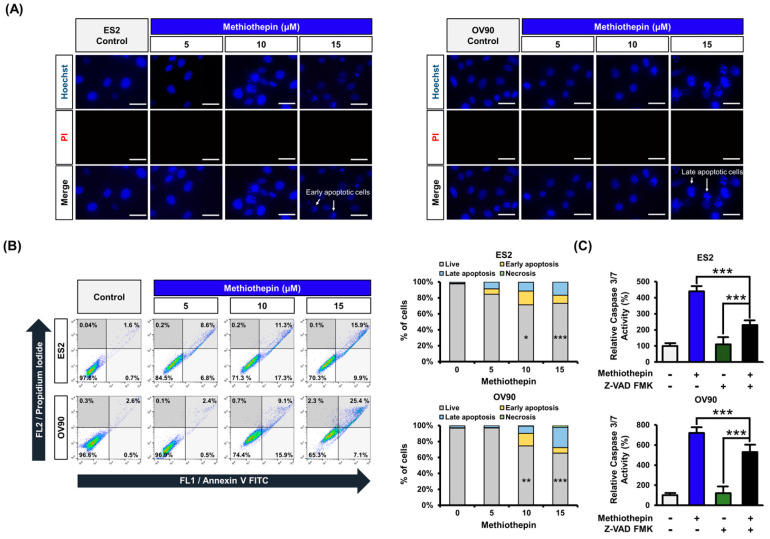
Regulation of cell death by methiothepin in ES2 and OV90 cells. (**A**) Hoechst (blue) and PI (red) staining was performed for cell death analysis in ES2 and OV90 cells. Scale bar indicates 40 μm. (**B**) Annexin V and PI staining were used to measure the death of ES2 and OV90 cells following methiothepin treatment. The percentage of cell distribution observed by flow cytometry was determined for the apoptotic pattern in each quadrant. The lower left quadrant corresponds to live cells, the upper left, to necrosis, the lower right, to early apoptosis, and the upper right, to late apoptosis. (**C**) The activity of caspase 3/7 in ES2 and OV90 cells treated with methiothepin was measured following the treatment of the cells with the pan-caspase inhibitor Z-VAD FMK. Results are expressed as the means ± SDs of three independent experiments. The asterisks indicate statistically significant differences compared to the control (* *p* < 0.05; ** *p* < 0.01; *** *p* < 0.001).

Next, to investigate the effect of methiothepin on cell death, ovarian cancer cells were stained with Hoechst and PI and microscopically imaged (Figure 2A). The blue fluorescent Hoechst dye stains the condensed chromatin of apoptotic cells brighter than that of normal cells. PI stains dead cells and gives them a red fluorescence. Following methiothepin treatment for 24 h at various doses (0, 5, 10, and 15 μM), staining with PI was barely observed in ES2 and OV90 cells, whereas Hoechst staining of condensed chromatin gradually increased following methiothepin treatment. These results suggest that methiothepin-treated ovarian cancer cells are undergoing the apoptotic process. When analyzing the staining pattern, it was confirmed that early apoptosis was mainly observed in ES2 cells and late apoptosis was observed in OV90 cells. To clarify that methiothepin induces apoptosis in ovarian cancer cells, we treated ovarian cancer cells with different doses of methiothepin (0, 5, 10, and 15 μM) for 24 h, followed by Annexin V and PI staining (Figure 2B). As a result, the number of normal cells decreased following methiothepin treatment, and the number of apoptotic cells was elevated dose-dependently. The proportion of ES2 cells in late apoptosis (upper right quadrant) increased from 1.6% in the control group to 15.9% in the 15 µM methiothepin treatment group. The percentage of OV90 cells undergoing late apoptosis increased from 2.6% to 25.4% with methiothepin treatment. Methiothepin also induced activation of caspase 3/7, which belongs to the apoptotic pathway (Figure 2C). Z-VAD FMK is a pan-caspase inhibitor that can suppress apoptosis by binding to the catalytic site of caspase proteases. Combination treatment with Z-VAD FMK reduced the activation of caspase 3/7 by methiothepin. These results suggest that methiothepin promotes apoptotic cell death in ovarian cancer cells. 

**Figure 3:**
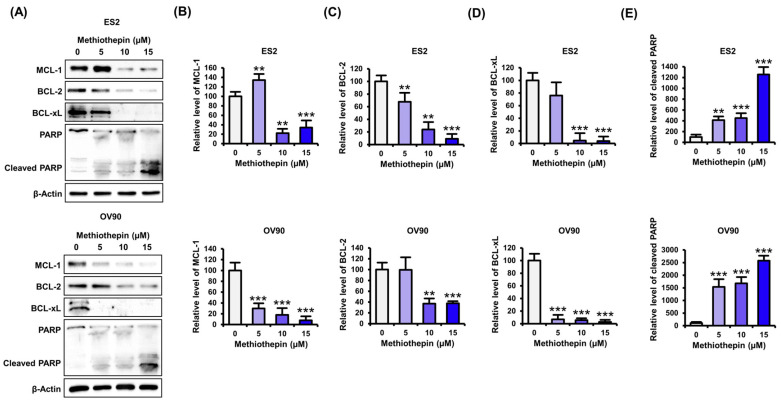
Effects of methiothepin on the expression of intrinsic apoptosis regulators in ES2 and OV90 cells. (**A**) Expression of apoptosis-related proteins following methiothepin treatment in ES2 and OV90 cells was analyzed by western blotting. (**B**–**E**) In order to quantify the expression of (**B**) MCL-1, (**C**) BCL-2, (**D**) BCL-xL, and (**E**) cleaved PARP in ES2 and OV90 cells following treatment with methiothepin, the intensity of the bands corresponding to each protein was normalized to the β-actin band intensity. The asterisks indicate statistically significant differences compared to the control (** *p* < 0.01; *** *p* < 0.001).

We performed western blotting to analyze the expression changes in anti-apoptotic proteins following methiothepin treatment in ovarian cancer cells (Figure 3A). The expression of MCL-1, an anti-apoptotic protein belonging to the BCL-2 family, was significantly reduced with methiothepin treatment (Figure 3B). In addition, the expression of BCL-2 also decreased with rising doses (0, 5, 10, and 15 μM) of methiothepin (Figure 3C). Methiothepin also inhibited the expression of BCL-xL in ES2 and OV90 cells, suggesting the progression of mitochondrion-dependent apoptosis (Figure 3D). Furthermore, the induction of PARP cleavage by methiothepin implies that the cell death pathway is active in ES2 and OV90 cells (Figure 3E). These results confirm that methiothepin can activate apoptotic pathways by inhibiting the activity of anti-apoptotic proteins in ovarian cancer cells.

### 3.3. Methiothepin Induces Mitochondrial Disruption in Ovarian Cancer Cells

**Figure 4:**
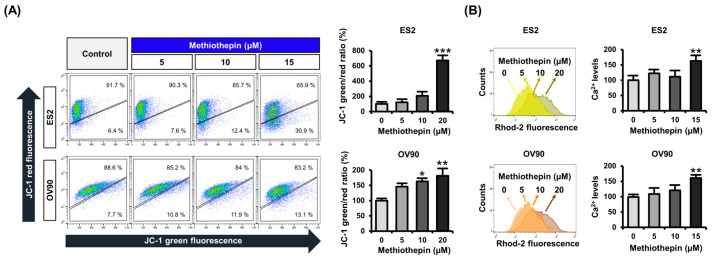
Effect of methiothepin on mitochondrial membrane potential (ΔΨ) and mitochondrial Ca^2+^ levels in ES2 and OV90 cells. (**A**) The change in ΔΨ by methiothepin in ES2 and OV90 cells was analyzed by JC-1 staining. The degree of depolarization of the mitochondrial membrane was determined based on JC-1 green/red ratio. (**B**) The change of mitochondrial Ca^2+^ levels in ES2 and OV90 cells following methiothepin treatment was analyzed by staining with the Rhod-2 dye. Results are expressed as the means ± SDs of three independent experiments. The asterisks indicate statistically significant differences compared to the control (* *p* < 0.05; ** *p* < 0.01; *** *p* < 0.001).

Alterations in the expression of proteins belonging to the intrinsic apoptosis pathway in methiothepin-treated cells suggest that methiothepin may induce mitochondrial disorders in ovarian cancer cells. In ovarian cancer cells, the mitochondrial membrane was depolarized dose-dependently when treated with methiothepin (0, 5, 10, and 15 μM) (Figure 4A). An increased loss of ΔΨ (JC-1 green/red ratio) in ES2 and OV90 cells reinforces that methiothepin causes mitochondrion-dependent apoptosis. The ratio of JC-1 green/red fluorescence intensity by 5, 10, and 20 μM methiothepin in ES2 cells was calculated to be 0.08, 0.14, and 0.47 (*p* < 0.001), respectively, while the control showed a value of 0.07. In OV90 cells, the JC-1 green/red fluorescence intensity by 0, 5, 10, and 20 μM methiothepin was 0.09, 0.13, 0.14 (*p* < 0.05), and 0.16 (*p* < 0.01). Moreover, we identified changes due to methiothepin in mitochondrion-specific Ca^2+^ levels through Rhod-2 staining. Methiothepin significantly increased the mitochondrial Ca^2+^ concentrations in ES2 and OV90 cells (Figure 4B). These results suggest that methiothepin can cause mitochondrial disruption leading to apoptosis in ovarian cancer cells.

### 3.4. Methiothepin Interferes with Mitochondrion-Mediated Metabolic Status in Ovarian Cancer Cells

**Figure 5:**
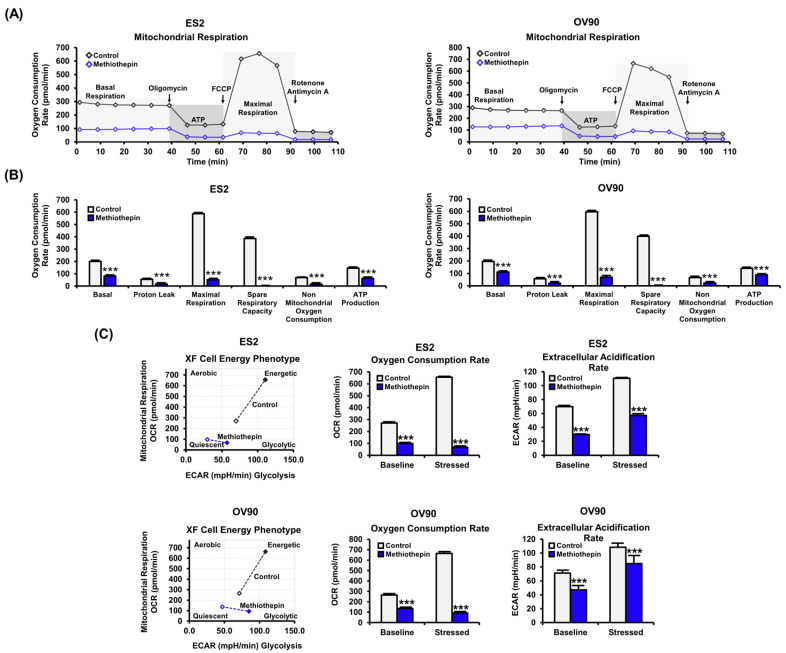
Metabolic profiling following methiothepin treatment in ES2 and OV90 cells. (**A**) The metabolic profiling changes in ES2 and OV90 cells following methiothepin treatment were analyzed by seahorse analysis with oligomycin, FCCP, Antimycin A, and Rotenone treatment. (**B**) Basal OCR, proton leak, maximal respiration, and ATP production were analyzed as the metabolic parameters in ES2 and OV90 cells in response to treatment with methiothepin. (**C**) The energy phenotypes in ES2 and OV90 cells were obtained by seahorse assay following treatment with methiothepin. In ES2 and OV90 cells, oxygen consumption rate and extracellular acidification rate under baseline (open marker) and stressed (closed marker) conditions following methiothepin treatment were analyzed. Results are expressed as the means ± SDs of three independent experiments. The asterisks indicate statistically significant differences compared to the control (*** *p* < 0.001).

Next, we analyzed the metabolic profile in response to methiothepin in ovarian cancer cells. We measured the oxygen consumption rate (OCR) by treatment with 15 μM of methiothepin for 24 h, followed by treatment with oligomycin, which inhibits ATP synthase, FCCP, which is an uncoupler of mitochondrial oxidative phosphorylation, and antimycin A and rotenone, which suppress mitochondrial electron transport in ES2 and OV90 cells (Figure 5A). The methiothepin-induced reduction of OCRs reflects the suppression of mitochondrial oxidative phosphorylation in the cancer cells, suggesting that electron transport chain activity was significantly decreased by methiothepin, leading to energy deprivation in ES2 and OV90 cells. In accordance with OCR inhibition, overall parameters for mitochondrial respiratory capacity were altered by methiothepin, and this supports the explicit modification of the metabolic landscape by methiothepin in cancer cells (Figure 5B). Beyond mitochondrial respiration, energy phenotypic changes imply glycolytic alterations by methiothepin in ES2 and OV90 cells, suggesting that methiothepin induced comprehensive metabolic disruption in ovarian cancer cells, contributing to an anti-proliferative effect (Figure 5C).

### 3.5. Methiothepin Regulates the Expression of Endoplasmic Reticulum Stress (ER)-Related Proteins in Ovarian Cancer Cells

**Figure 6:**
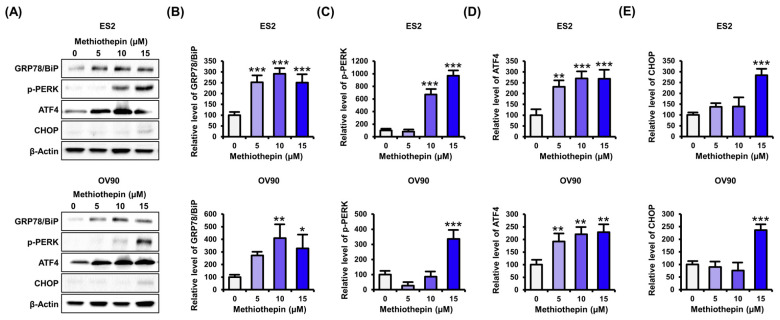
Effects of methiothepin on the expression of ER stress-related proteins in ES2 and OV90 cells. (**A**) Expression of ER stress-related proteins following methiothepin treatment in ES2 and OV90 cells was analyzed through western blotting. (**B**–**E**) In order to quantify the expression of (**B**) GRP78/Bip, (**C**) p-PERK, (**D**) ATF4, and (**E**) CHOP in ES2 and OV90 cells following treatment with methiothepin, the intensity of the band corresponding to each protein was normalized to the β-actin band intensity. The asterisks indicate statistically significant differences compared to the control (* *p* < 0.05; ** *p* < 0.01; *** *p* < 0.001).

Next, thinking that mitochondrial disruption by methiothepin may be derived from ER stress, we analyzed the expression of ER stress-related proteins following methiothepin treatment in ovarian cancer cells. Western blot analysis revealed that methiothepin dose-dependently (0, 5, 10, and 15 μM) increased the expression of ER stress-related proteins in ES2 and OV90 cells (Figure 6A). The expression of GRP78/Bip, a chaperone located in the lumen of the ER and a target of ER stress response, was significantly increased by methiothepin (Figure 6B). PERK, an ER transmembrane protein that promotes the expression of pro-apoptotic genes in response to ER stress, was also activated by methiothepin in ES2 and OV90 cells (Figure 6C). ATF4 is a protein that stimulates translation by PERK, and its expression was increased by treatment with methiothepin in ES2 and OV90 cells (Figure 6D). ATF4 induces transcription of the pro-apoptotic protein CHOP. Our results confirm that methiothepin also elevates CHOP expression (Figure 6E). These results imply that methiothepin can lead to cell death by inducing ER stress in ovarian cancer cells.

### 3.6. Methiothepin Interferes with the Development of the Vascular System in Zebrafish Embryos

**Figure 7:**
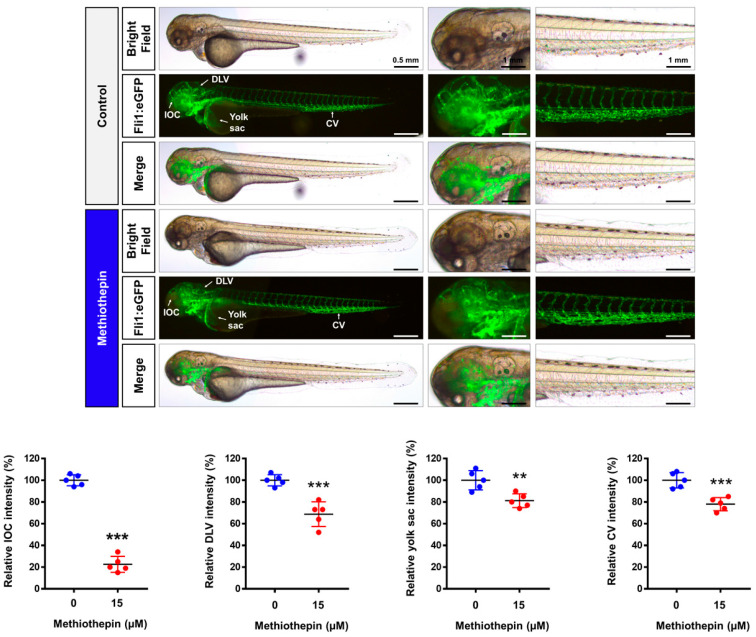
Impairment of vasculogenesis by methiothepin exposure in zebrafish embryos. Fluorescence images of transgenic (fli1:eGFP) zebrafish embryos after treatment with methiothepin for 48 h were captured by fluorescent microscopy. Quantification of fluorescence intensity in the vascular structures of the inner optic circle (IOC), dorsal longitudinal vein (DLV), yolk sac, and caudal vein (CV) of zebrafish embryos following methiothepin exposure was performed using ImageJ software. The asterisks indicate statistically significant differences compared to the control (** *p* < 0.01; *** *p* < 0.001). Scale bar represents 0.5 mm (the first vertical panels) and 1 mm (the second and third vertical panels).

An important feature to understand among the mechanisms of cancer development is the development of the vascular system around the tumor. Angiogenesis is one of the hallmarks of cancer and is the basis for tumor growth and invasion, leading to metastasis to other organs [14]. Embryos of the transgenic zebrafish line (fli1:eGFP) are a very valuable model for studying vascular development disorders; these embryos express eGFP under the control of the fli1 promoter, which is essential for vascular development [15]. Fluorescence microscopy revealed that methiothepin treatment for 48 h in embryos inhibited fluorescence signals in the inner optic circle, dorsal longitudinal vein, yolk sac, and caudal vein, compared to those of the solvent-treated control (Figure 7). These results suggest that methiothepin may have anti-angiogenic effects in the tumor microenvironment and contribute anti-metastatic functions in ovarian cancer cells. 

### 3.7. Methiothepin Enhances the Anti-Cancer Effects Induced by Paclitaxel

**Figure 8:**
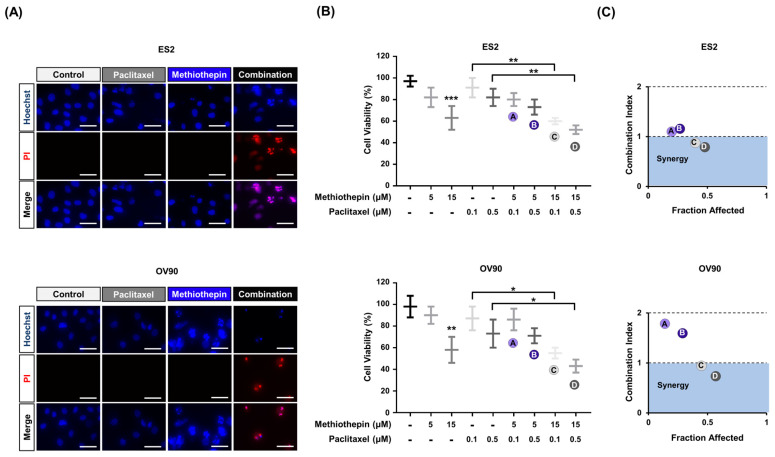
Synergistic effects of methiothepin treatment on paclitaxel sensitivity in ES2 and OV90 cells. (**A**) Hoechst (blue) and PI (red) staining analysis of ES2 and OV90 cells after combination treatment with methiothepin and paclitaxel. Scale bar indicates 40 μm. (**B**) Analysis of the viabilities of ES2 and OV90 cells revealed that methiothepin and paclitaxel are synergistic in inhibiting the viability of these cells. (**C**) The synergistic effects of the combination of methiothepin and paclitaxel on the viability of ES2 and OV90 cells were calculated based on the combination index (CI) and fraction affected (FA) using the CompuSyn software. Results are expressed as the means ± SDs of three independent experiments. The asterisks indicate statistically significant differences compared to the control (* *p* < 0.05; ** *p* < 0.01; *** *p* < 0.001).

Finally, the combined treatment of methiothepin and paclitaxel was performed for 24 h to see if methiothepin could show synergism with the anti-cancer effects of paclitaxel, the standard treatment regimen for ovarian cancer. After that, Hoechst and PI dye were used to evaluate the cell death in the ES2 and OV90 cells (Figure 8A). As shown in Figure 2A, staining with PI was not evident following the treatment with methiothepin alone. Paclitaxel alone also did not induce fluorescence expression for PI staining in ES2 and OV90 cells. However, the combination treatment with both induced the red fluorescence of PI. To clarify the synergistic effect on ovarian cancer, we measured cell viability after treatment with the combination of methiothepin and paclitaxel for 24 h (Figure 8B). We found that 0.1 μM or 0.5 μM of paclitaxel did not significantly inhibit cell viability. In ES2 and OV90 cells treated with 5 μM of methiothepin in combination with paclitaxel, there was no significant decrease in cell viability, but 15 μM of methiothepin decreased the viability of cells, compared to that of cells treated with paclitaxel alone. These results were verified by confirming that the combination index was less than one when 15 μM methiothepin (C and D) was combined with paclitaxel in ES2 and OV90 cells (Figure 8C). These results suggest that methiothepin has the potential to be used as an adjuvant to increase the efficiency of paclitaxel-based chemotherapy.

## 4. Discussion

Over the past few decades, advances in surgical techniques and chemotherapy have improved the survival of ovarian cancer patients. However, most patients receiving paclitaxel and platinum-based drugs experience relapse [4]. Mechanisms determining the prognosis of ovarian cancer are not yet clear, but various hormones, growth factors, and neurotransmitters are reported to activate receptor-mediated growth signals in ovarian cancer cells [5,16,17]. Therefore, antagonists of receptors that contribute to the activation of growth signals have potential as therapeutic adjuncts to ovarian cancer [18,19]. 

Neurotransmitters are representative extracellular signals that induce the activation of GPCRs, which mediate extensive cellular signaling pathways [20]. It is well-known that several types of neurotransmitters including acetylcholine, epinephrine, dopamine, and serotonin have effects on cancer. Serotonin signaling is activated by the binding of serotonin to seven types of receptor families. 5-HT_2A_, 5-HT_2B_, and 5-HT_2C_ bind to Gαq/11 protein to activate phospholipase C, which induces intracellular Ca^2+^ release. 5-HT_4_, 5-HT_6_, and 5-HT_7_ receptors bind to Gαs to convert ATP to cAMP, interacting with protein kinase A (PKA) to regulate the intracellular Ca^2+^ levels [21]. Notably, expression of the 5-HT_2A_ receptor is higher in ovarian cancer cells than in normal cells. Some studies have reported that antidepressants are responsible for carcinogenesis [22,23], while other studies have shown that some antidepressants have anti-proliferative effects on cancer cells [24]. This evidence suggests that modulators for serotonin not only function as antidepressants, but can also be used as an effective regimen for cancer therapy. 

In this study, it was verified that methiothepin, a serotonin receptor antagonist, can reduce the viability and proliferation of ovarian cancer cells. Thus far, aside from inhibiting the activity of the serotonin receptor, the physiological activity of methiothepin is unclear. In the medial preoptic area of estrogen-treated ovariectomized rats, methiothepin microinjection lowers the level of plasma luteinizing hormone, suggesting that serotonin may promote secretion through 5HT_1_ [25]. In bovine ovarian follicles, methiothepin antagonizes the release of electrically stimulated transmitters by serotonin [26]. Although our previous results in prostate cancer cells suggest that methiothepin has anti-cancer effects, the mechanism of its action in ovarian cancer was first identified in this study [12]. In ovarian cancer cells, methiothepin is thought to induce cell death through apoptotic pathways that mediate caspase activation. Our results also revealed that the intrinsic apoptotic pathway, which is mediated by methiothepin, may appear with disorders of organelles such as the mitochondrion or ER. Methiothepin also stimulates mitochondrion-specific Ca^2+^ influx in ovarian cancer cells.

The results of previous studies suggest that compounds that modulate serotonin activity can cause metabolic disorders with the dysfunction of organelles. SSRIs may affect cancer progression; however, inconsistent results have been observed in this regard [22,27]. Fluoxetine, the most widely used clinical antidepressant and SSRI, inhibits tumor growth in various types of cells including colorectal carcinoma and melanoma cells [28,29]. Fluoxetine increases intracellular Ca^2+^ levels in immune cells and nervous cells [30,31]. This evidence suggests that fluoxetine regulates the pathway leading to the activation of the IP3 receptor (IP_3_R) present on the ER membrane through the activation of phospholipase C. Mitochondria are central organelles that mediate Ca^2+^-induced cell death. Ca^2+^ homeostasis is essential for cell survival and Ca^2+^ overload into mitochondria leads to cell death by necrosis or apoptosis [32]. Fluoxetine accumulates predominantly in mitochondria, implying that fluoxetine-induced increase in Ca^2+^ levels results in mitochondrion-dependent cell death [33]. SSRIs also cause metabolic disorders that result from reduced oxygen consumption and disruption of mitochondrial ATP production in human cells [34]. These results are consistent with the results of this study, which stated that methiothepin can not only induce mitochondrial dysfunction, but also cause metabolic disorders such as disrupted ATP production and suppression of the OCR in ovarian cancer.

Drug resistance induced by chemotherapy is a major obstacle in the treatment of ovarian cancer. It is assumed that there are several possible mechanisms underlying the development of chemoresistance, one of which is metabolic alterations including that of glycolytic and lipid pathways [35]. In chemotherapy-resistant ovarian cancer cells, the alteration of mitochondrial function and metabolic status has been verified by enhanced ATP levels and lower OCR in the mitochondria [36]. As cells acquire drug resistance, they adapt to the change in cellular respiration [37]. The dependence of cancer cells on aerobic glycolysis for their high energy demands is a target for therapeutic approaches [38]. Cellular respiration is a catabolic reaction that yields ATP. Unlike normal cells, cancer cells rely on a process with low energy efficiency, and this characteristic is important in distinguishing metabolically malignant tumors from normal cells [37]. Benign cells use the TCA cycle and oxidative phosphorylation as the main sources of ATP, whereas malignant cells utilize glycolysis as an energy source [39]. The AMPK system is a cell energy state sensor that is activated by an increase in the AMP:ATP ratio caused by metabolic stresses that disrupt ATP production. Mitochondrial metabolic targeting, which makes use of the difference in energy utilization between normal and cancer cells, is considered an important therapeutic approach in ovarian cancer [40]. 

To overcome chemoresistance in cancer, it is necessary to understand the various pathways of cell death. Mcl-1 is an anti-apoptotic protein of the Bcl-2 family and is important for the development and maintenance of the immune and nervous systems [41,42]. Proteins belonging to the Bcl-2 family are responsible for mitochondrial outer membrane permeabilization and are important regulators of the intrinsic apoptosis pathway. The expression of these proteins has been proven to be significantly inhibited by methiothepin exposure in ovarian cancer. ER stress is also considered to be a target that can effectively promote the death of cancer cells following chemotherapy [43]. The imbalance of intracellular energy or Ca^2+^ levels leads to the accumulation of unfolded proteins [44]. In order to maintain homeostasis, the pathway of protein activation by ER stress is represented by complex and diverse routes. In this study, we investigated the regulatory mechanisms underlying the action of methiothepin on ER stress-related proteins ranging from GRP78/Bip, an ER chaperone, to CHOP, which activates caspase to induce apoptosis. Crucially, the combination treatment with methiothepin is expected to further improve the anti-cancer effect of paclitaxel. In addition, the anti-angiogenic action of methiothepin in zebrafish embryos suggests that methiothepin may contribute to improving the tumor microenvironment in ovarian cancer. In ovarian cancer, like any carcinoma, angiogenesis is an important therapeutic target because it is crucial for tumor invasion and metastasis [45].

## 5. Conclusions

**Figure 9:**
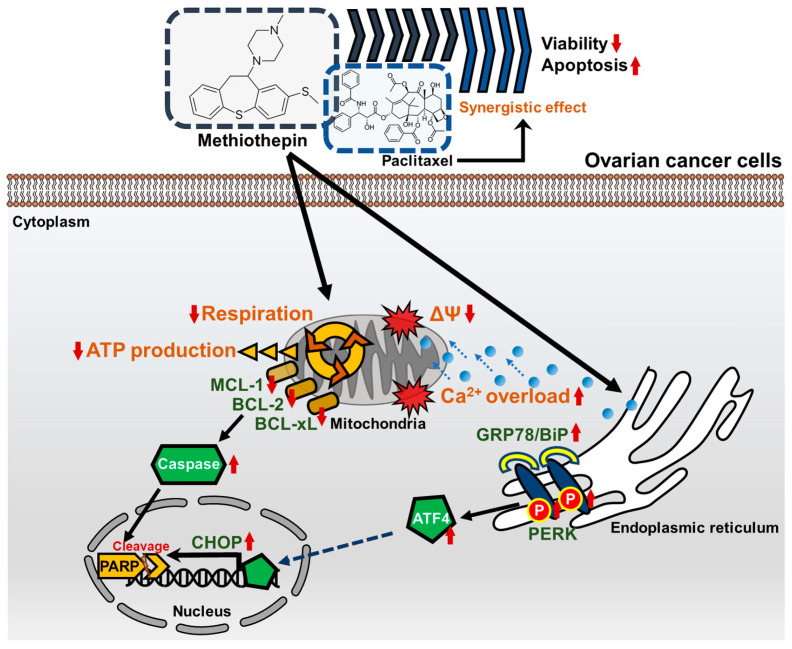
Schematic diagram of the methiothepin-associated anti-cancer mechanisms in ovarian cancer cells. In ovarian cancer cells, methiothepin induces depolarization in the mitochondrial membrane (also known as mitochondria membrane potential, ΔΨ) and promotes the influx of Ca^2+^. Metabolic disorders caused by methiothepin are indicated by a decrease in mitochondrial respiration and adenosine triphosphate (ATP) production. The expression of proteins belonging to the BCL-2 family are inhibited by methiothepin, which leads to the cleavage of poly (ADP-ribose) polymerase (PARP), along with the activation of caspases. Meanwhile, methiothepin increases the expression of ER stress-related proteins and finally induces the expression of C/EBP homologous protein (CHOP), which leads to apoptosis. Methiothepin improves the anti-cancer effect of paclitaxel, greatly reducing the viability of ovarian cancer cells.

Taken together, for the first time, this study identified that methiothepin inhibits the viability of, and induces apoptosis in, ovarian cancer cells, as illustrated in Figure 9. Moreover, methiothepin regulates the expression of proteins associated with intrinsic apoptotic pathways and ER stress. By verifying the induction of mitochondrial and metabolic disorders by methiothepin, specific mechanisms underlying the action of methiothepin in human cancer cells were confirmed. Furthermore, the effect of methiothepin, which improves the anti-cancer effects of paclitaxel, suggests its potential to serve as a potential therapeutic adjunct in ovarian cancer treatment.
